# Efficacy of cadonilimab in combination with nimotuzumab and AG regimen in TMB-H/KRAS wild-type advanced pancreatic cancer: a case report

**DOI:** 10.3389/fimmu.2025.1652827

**Published:** 2025-09-30

**Authors:** Zhiwei Tang, Jiegang Hu, ZhongShi He, Dongdong Zhang

**Affiliations:** Department of Oncology, Xiangyang No. 1 People’s Hospital, Hubei University of Medicine, Xiangyang, China

**Keywords:** advanced pancreatic cancer, tumor mutational burden (TMB-H), KRAS wild-type, cadonilimab, nimotuzumab

## Abstract

**Background:**

Advanced pancreatic cancer carries a dismal prognosis. Current chemotherapy provides limited survival benefit while causing substantial toxicity. Despite numerous trials, combining gemcitabine with other cytotoxic or targeted agents has not significantly improved outcomes, highlighting the urgent need for novel therapeutic strategies.

**Case presentation:**

This report describes a 56-year-old male diagnosed with stage IVB pancreatic tail adenocarcinoma, characterized by a high tumor mutational burden (TMB-H) and a KRAS wild-type status. The patient showed a significant therapeutic response and an improved quality of life after receiving a novel four-drug combination regimen. The treatment included cadonilimab (a PD-1/CTLA-4 bispecific antibody), nimotuzumab (an EGFR monoclonal antibody), albumin-bound paclitaxel, and gemcitabine. After two cycles, the primary pancreatic lesion reduced by 37%, and there was substantial shrinkage of hepatic metastases. Continued treatment maintained partial remission (PR), with progression-free survival (PFS) lasting over seven months and manageable toxicity.

**Conclusion:**

This case highlights the potential of the combination of cadonilimab, nimotuzumab, albumin-bound paclitaxel, and gemcitabine as an effective, low-toxicity treatment option for patients with TMB-H/KRAS wild-type advanced pancreatic cancer.

## Introduction

Pancreatic cancer is one of the most aggressive and fatal malignancies, with a poor prognosis (1). About 80% of patients are diagnosed at advanced stages, either with locally advanced disease or widespread metastases, resulting in a median overall survival of less than 12 months (2). Despite using first-line chemotherapeutic regimens like FOLFIRINOX (oxaliplatin/irinotecan/5-FU/leucovorin) and the combination of albumin-bound paclitaxel with gemcitabine (AG regimen), survival outcomes remain limited (3, 4). These treatments are also often associated with significant hematologic and neurotoxicities, further reducing their clinical utility. Therefore, there is an urgent need for more effective and better-tolerated therapeutic strategies for patients with advanced pancreatic cancer.

Recently, molecularly guided precision therapy has emerged as a promising approach for treating advanced pancreatic adenocarcinoma (PDAC). A key feature of pancreatic cancer is the overexpression of epidermal growth factor receptor (EGFR), observed in 30% to 70% of patients, both in primary and metastatic lesions (5). While the combination of cetuximab, an EGFR-targeting monoclonal antibody, with gemcitabine failed to show superior efficacy compared to gemcitabine monotherapy (6), other studies suggest that combining nimotuzumab, another EGFR monoclonal antibody, with gemcitabine can significantly improve both overall survival (OS) and progression-free survival (PFS) in patients with KRAS wild-type PDAC (2). This suggests that EGFR-targeting therapies may be effective, particularly in patients with specific genetic profiles, such as KRAS wild-type status.

Immune checkpoint inhibitors, such as PD-1/PD-L1 inhibitors and CTLA-4 inhibitors, have been widely studied in PDAC. Their success has been limited to subpopulations with high tumor mutational burden (TMB-H) or high microsatellite instability (MSI-H), where immune responses are more likely to be effective (7, 8). However, factors such as sparse CD8+ T-cell infiltration, dense stromal barriers, and immunosuppressive elements in the general pancreatic cancer population contribute to the limited efficacy of monotherapy immune checkpoint inhibitors (9, 10). This reflects the “immunologically cold” nature of pancreatic cancer, characterized by a highly immunosuppressive tumor microenvironment (TME) (11). The failure of single-agent immunotherapies to overcome this hostile environment underscores the need for combination therapies that can target multiple immune pathways and tumor mechanisms.

In response to these challenges, we present a clinically promising case of advanced PDAC with dual actionable biomarkers: TMB-H and KRAS wild-type status. The patient received a novel quadruple-modality regimen consisting of cadonilimab (a dual PD-1/CTLA-4 inhibitor), nimotuzumab (an EGFR monoclonal antibody), and the AG regimen.

## Case report

**Figure 1:**
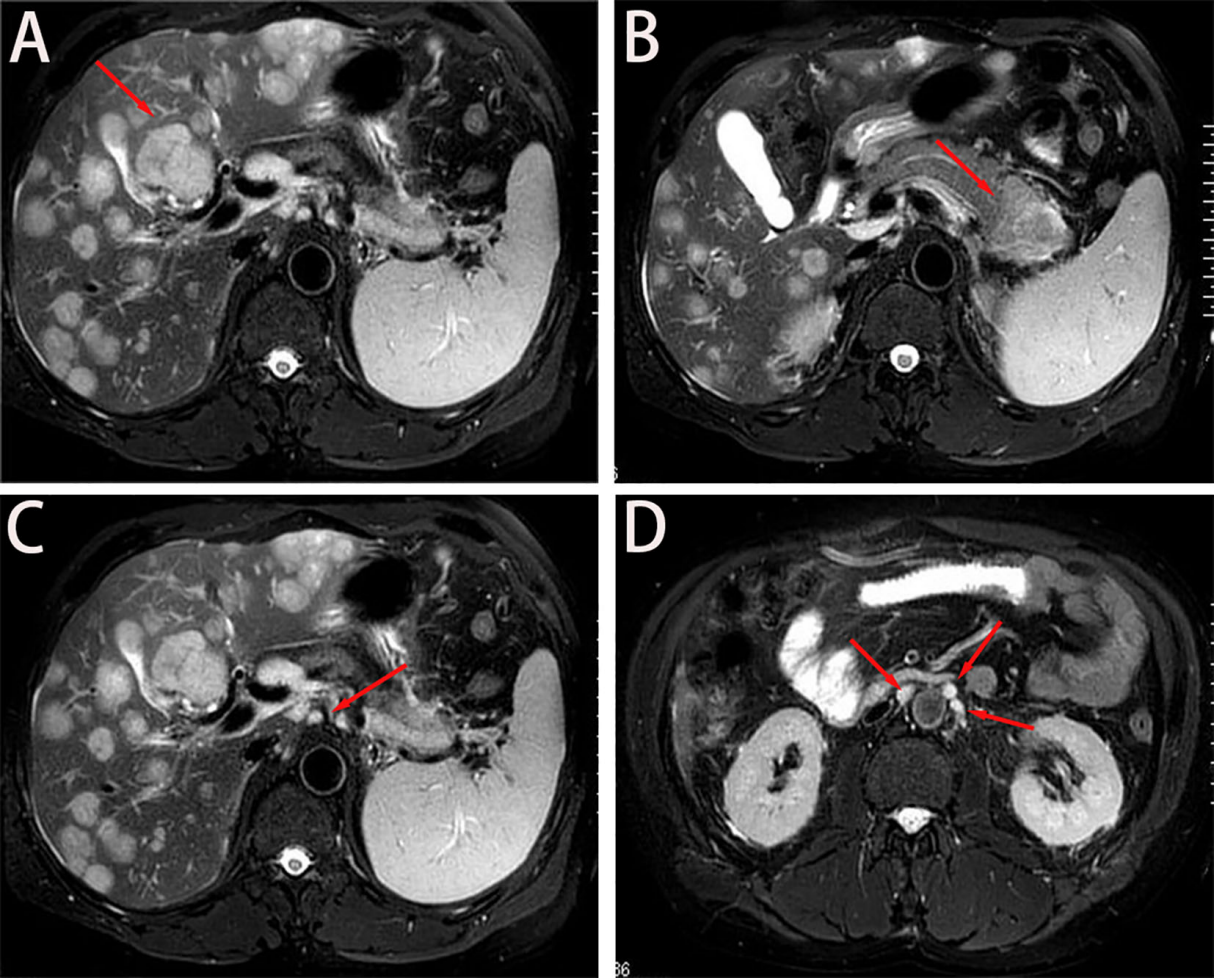
Baseline imaging findings of metastatic pancreatic adenocarcinoma. **(A, B)** Axial T2- weighted MRI demonstrates: **(A)** Diffuse hepatic metastases (arrows) with characteristic hyperintense signal; **(B)** A 4.7 × 3.7 cm² heterogeneously enhancing primary mass (asterisk) in the pancreatic tail. **(C)** Coronal view reveals metastatic involvement of the hepatic hilar lymph nodes (arrowheads), showing irregular margins and diffusion restriction. **(D)** Sagittal reconstruction identifies multiple enlarged retroperitoneal lymph nodes (circles) along the para-aortic region, consistent with metastatic spread.

**Figure 2:**
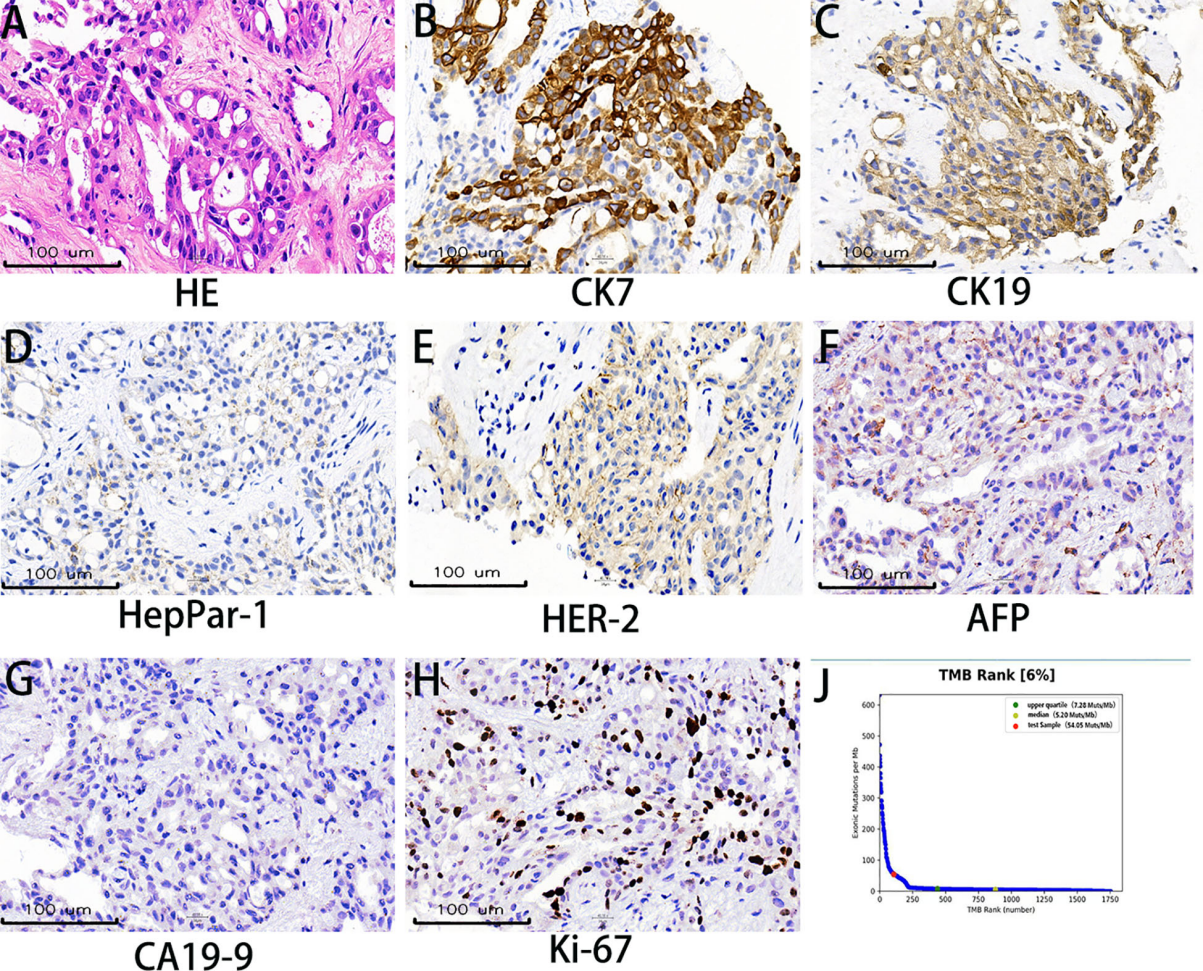
Histopathological and molecular characteristics. **(A)** Hematoxylin and eosin (H&E) staining demonstrates infiltrating tumor cells exhibiting polygonal to columnar morphology with moderate cytoplasm, nuclear pleomorphism, and invasive growth patterns. **(B, C)** Immunohistochemical (IHC) analysis reveals strong cytoplasmic expression of CK7 and CK19, supporting a pancreaticobiliary or upper gastrointestinal tract origin. **(D)** HepPar-1 negativity effectively excludes hepatocellular carcinoma and hepatoid adenocarcinoma in the differential diagnosis. **(E)** HER-2 immunohistochemistry shows incomplete membranous staining (1+), indicating weak positivity. **(F)** Absence of AFP expression rules out metastatic hepatoid differentiation or germ cell tumors. **(G)** CA19–9 negativity, observed in approximately 10% of pancreatic adenocarcinomas, provides additional diagnostic context. **(H)** Ki-67 nuclear staining demonstrates a proliferative index of 10%. **(I)** Next-generation sequencing revealed a high tumor mutational burden of 54.05 mutations/mega base.

**Figure 3:**
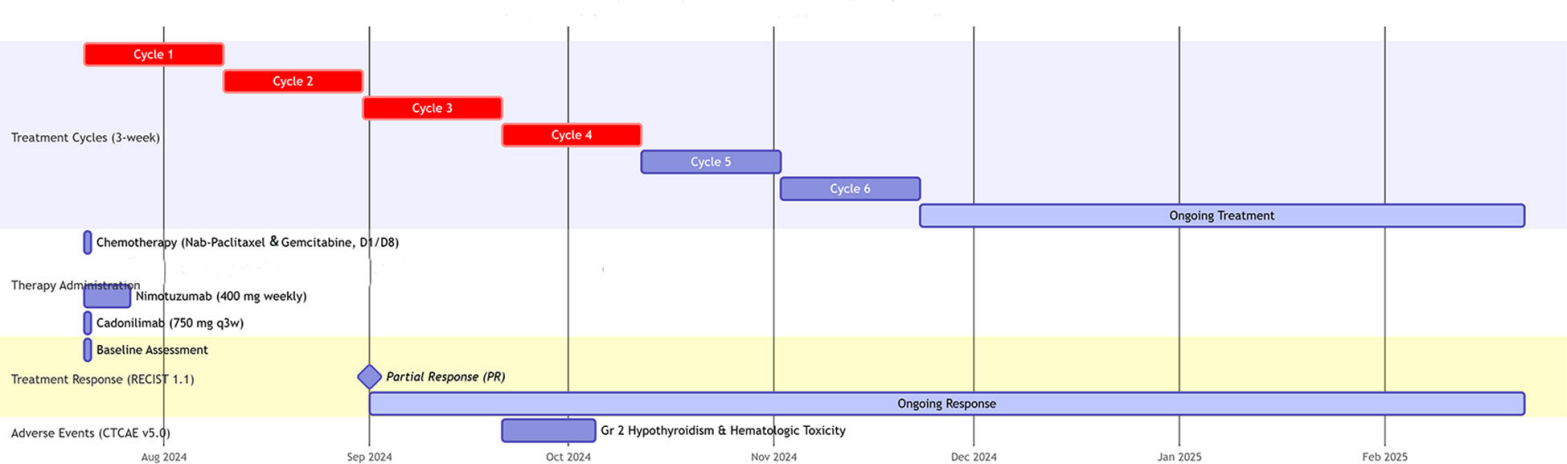
Swimmer plot of treatment timeline and clinical course. ECOG PS, Eastern Cooperative Oncology Group Performance Status; D, day; q3w, every 3 weeks; RECIST 1.1, Response Evaluation Criteria in Solid Tumors version 1.1; PR, Partial Response; CTCAE, Common Terminology Criteria for Adverse Events; Gr, Grade.

A 56-year-old male with well-controlled hypertension presented with a one-week history of epigastric pain and was admitted on July 12, 2024. Initial evaluation showed mild leukocytosis and elevated tumor markers (AFP: 9.22 ng/mL [normal: 0-8.7]; CEA: 78.4 ng/mL [normal: 0-7.2]). Contrast-enhanced MRI (July 14, 2024) identified a 4.7 × 3.7 cm malignant lesion in the pancreatic tail with synchronous hepatic metastases and hilar/retroperitoneal lymphadenopathy (**Figure 1**). Percutaneous liver biopsy (July 15, 2024) confirmed metastatic adenocarcinoma (**Figure 2A**), with immunohistochemistry showing CK7 (+), CK19 (+), HepPar-1 (–), HER-2 (1+), AFP (-), CA19-9 (-) and a Ki-67 index of 10% (**Figures 2B–H**). Molecular profiling revealed a high tumor mutational burden (TMB) of 54.05 mutations per mega base (TMB-H) (**Figure 2J**). The tumor was microsatellite stable (MSS) and lacked BRCA1/BRCA2, BRAF, and KRAS mutations. The patient was diagnosed with stage IVB PDAC (AJCC 8th edition) with TMB-H/MSS/KRAS wild-type features. The patient, with an ECOG performance status of 1 and adequate organ function, began quadruple therapy on July 20, 2024. The chemotherapy regimen included nab-paclitaxel (200 mg on days 1 and 8) combined with gemcitabine (1.4 g on days 1 and 8). Nimotuzumab was administered for targeted therapy at 400 mg weekly, while the immunotherapy component included cadonilimab at a dose of 750 mg every three weeks (**Figure 3**).

**Figure 4:**
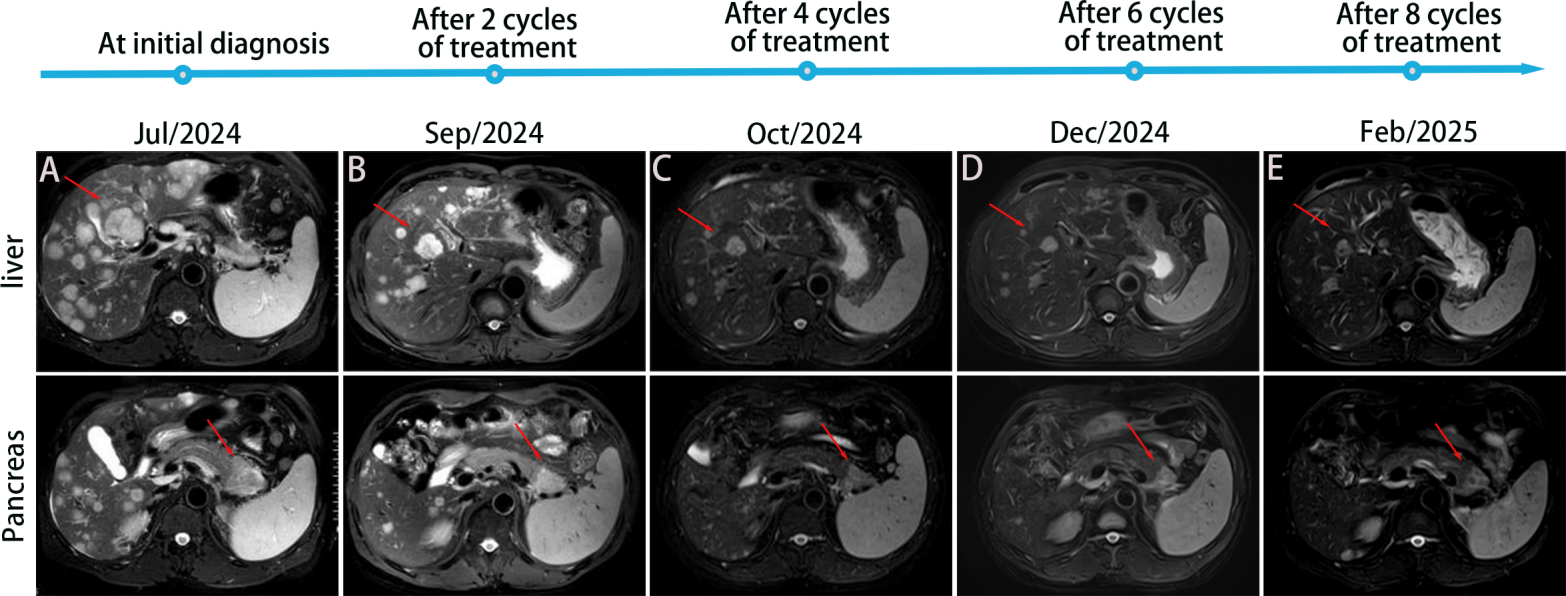
Treatment timeline and imaging follow-up. MRI T2-weighted images: **(A)** Diffuse intrahepatic metastatic foci before treatment (July 12, 2024), with a mass observed in the tail of the pancreas. **(B)** Intrahepatic metastatic foci and the pancreatic tail lesion significantly reduced and minimized after 2 cycles of treatment. **(C-E)** Further reduction and minimization of intrahepatic metastatic foci and pancreatic tail lesion after 4, 6, and 8 cycles of treatment, respectively.

After two cycles of treatment, imaging showed a 35% reduction in the primary lesion and significant regression of hepatic metastases, meeting the RECIST 1.1 criteria for partial response (PR). PR continued through cycles 4, 6, and 8. By February 21, 2025, additional shrinkage of the primary tumor was noted, with sustained hepatic response. PFS exceeded 7 months, and the patient experienced an improvement in quality of life. Treatment-related adverse events were limited to grade 2 hypothyroidism and hematologic toxicity, both of which resolved with supportive care. The detailed treatment timeline and imaging follow-up are shown in **Figure 4**.

## Discussion

Treating advanced PDAC remains a significant clinical challenge. This case presents an innovative therapeutic approach combining immune checkpoint inhibition, targeted therapy, and chemotherapy. This integrative approach resulted in sustained tumor regression with manageable toxicity, providing mechanistic insights into the synergy between dual immune checkpoint inhibition, EGFR targeting, and chemotherapy-induced immunogenic cell death.

Gemcitabine has long been the cornerstone of first-line therapy for advanced PDAC, providing consistent, albeit modest, survival benefits. However, the landmark MPACT trial demonstrated significant therapeutic advancement with the AG regimen, achieving superior median OS compared to gemcitabine monotherapy (8.7 vs. 6.6 months; HR = 0.72, 95% CI: 0.62–0.83; p<0.001) (4). While the FOLFIRINOX regimen showed comparable efficacy (OS: 11.1 vs. 6.8 months), its utility is limited by substantial toxicity, including grade ≥3 or severe neutropenia and diarrhea (12). In contrast, the AG regimen demonstrated a more favorable safety profile, with lower incidences of severe hematologic and neurotoxic effects. Based on these considerations, we selected the AG regimen as the chemotherapeutic backbone for our combination strategy, aimed at cytotoxic debulking and immunogenic modulation.

Nimotuzumab (Nimo) is a humanized IgG1 monoclonal antibody that specifically targets the extracellular domain of EGFR. It exerts antitumor effects by inhibiting ligand-dependent EGFR dimerization and downstream proliferative signaling in the RAS-MAPK pathway, as well as suppressing VEGF-mediated angiogenesis. Emerging evidence suggests that EGFR inhibition potentiates chemotherapy efficacy by normalizing tumor vasculature and enhancing drug delivery (13). Compared to other EGFR inhibitors, such as cetuximab, Nimo offers two major pharmacodynamic advantages. First, it has a significantly better safety profile, with fewer severe dermatologic toxicities compared to cetuximab (14). Second, Nimo exhibits more targeted biodistribution, with a higher tumor-to-plasma concentration ratio in EGFR-overexpressing tumors, enhancing its effectiveness at reaching the tumor site (14). The phase III NOTABLE trial validated these properties in PDAC, demonstrating significant survival benefits for Nimo plus gemcitabine compared to gemcitabine alone in KRAS wild-type patients (15). Given our patient’s KRAS wild-type status and Nimo’s dual capacity to disrupt oncogenic signaling and potentiate chemotherapy efficacy, we incorporated this agent into our therapeutic regimen.

Cadonilimab, a tetravalent PD-1/CTLA-4 bispecific antibody, engages both immune checkpoints via trans-binding while avoiding Fc receptor interactions, thereby minimizing cytokine release syndrome (IL-6/IL-8) and antibody-dependent cytotoxicity (16). This design provides a superior safety profile. The phase 1b/2 COMPASSION-03 trial demonstrated that cadonilimab monotherapy showed manageable toxicity and promising efficacy in advanced solid tumors (17). Notably, the phase 3 COMPASSION-16 trial validated the clinical value of cadonilimab in immunologically cold tumors, showing significant PFS and OS benefits when combined with first-line chemotherapy in persistent, recurrent, or metastatic cervical cancer (18). Given our patient’s advanced PDAC, multifocal hepatic metastases, and the established synergy between PD-1/CTLA-4 co-blockade and high mutational burden, cadonilimab was integrated into the AG-nimotuzumab backbone.

This therapeutic strategy exerts antitumor effects through four synergistic mechanisms. First, dual PD-1/CTLA-4 checkpoint blockade alleviates T-cell exhaustion by simultaneously counteracting PD-1-mediated suppression of T-cell effector functions and CTLA-4-driven inhibition of dendritic cell co-stimulatory signaling, promoting clonal expansion and functional reinvigoration of CD8+ cytotoxic T lymphocytes (19). Second, the high tumor mutational burden generates abundant neoantigens that synergize with dual checkpoint inhibition to enhance tumor immunogenicity. This is achieved through enhanced MHC class I antigen presentation, facilitating tumor-specific T-cell recognition and immunological synapse formation. Third, preclinical evidence suggests that CTLA-4 blockade may augment hepatic natural killer cell-mediated enrichment (20). Third, nimotuzumab potentially exerts a dual-sensitizing effect by not only chemosensitizing the tumor through inhibition of EGFR-driven proliferation and survival pathways, thereby potentiating the cytotoxicity of both gemcitabine and nab-paclitaxel, but also by reprogramming the immunosuppressive tumor microenvironment; this reversal of immune suppression may enhance T-cell function and consequently augment the efficacy of the PD-1/CTLA-4 bispecific antibody. Additionally, combining anti-PD-1 antibody immunotherapy with gemcitabine (GEM) enhances the immune response mediated by Th1 lymphocytes, M1 macrophages, and CD8+ T cells (21), which could be particularly beneficial for patients with liver metastasis, further enhancing overall therapeutic efficacy. A recent clinical trial revealed that dual PD-1/CTLA-4 inhibition achieved significantly better PFS compared to PD-1/PARP inhibitor combinations in platinum-sensitive advanced PDAC, validating the role of bispecific antibodies in maintenance therapy (22).

This study has several limitations that should be considered. First, as a single-case report, the findings lack generalizability and require validation in larger cohorts. Second, while we used the AG regimen, emerging data from the NAPOLI-3 trial (liposomal irinotecan/5-fluorouracil/leucovorin/oxaliplatin, NALIRIFOX) demonstrate sustained OS benefits at 12 and 18 months compared to AG. The potential efficacy of NALIRIFOX combined with nimotuzumab and cadonilimab remains unexplored. Third, the relatively short follow-up duration prevents assessment of long-term treatment-related toxicities and efficacy. Fourth, the optimal maintenance strategy (immune-nimotuzumab dual therapy vs chemo-immunotherapy) once the disease stabilizes, and the optimal duration of maintenance therapy, remain unknown. These unanswered questions highlight the need for prospective, randomized controlled trials to validate our preliminary observations and optimize therapeutic sequencing.

## Conclusion

In this study, we demonstrate that a novel quadruplet regimen combining the PD-1/CTLA-4 bispecific antibody (cadonilimab), EGFR inhibition (nimotuzumab), and chemotherapy (AG regimen) achieved promising antitumor activity in a patient with advanced PDAC characterized by KRAS wild-type status, high tumor mutational burden (TMB-H), and multifocal hepatic metastases. This study suggests that combining dual immune checkpoint blockade (PD-1/CTLA-4), EGFR inhibition, and chemotherapy may benefit molecularly selected advanced PDAC patients with KRAS wild-type and TMB-H status. Although requiring validation, these findings support the development of biomarker-driven phase II trials to evaluate this precision immuno-oncology approach.

## Data Availability

The original contributions presented in the study are included in the article/supplementary material. Further inquiries can be directed to the corresponding authors.

## References

[1] Halbrook CJ Lyssiotis CA Pasca di Magliano M Maitra A . Pancreatic cancer: Advances and challenges. Cell. (2023) 186:1729–54. doi: 10.1016/j.cell.2023.02.014, PMID: 37059070 PMC10182830

[2] Qin S Li J Bai Y Wang Z Chen Z Xu R . Nimotuzumab plus gemcitabine for K-ras wild-type locally advanced or metastatic pancreatic cancer. J Clin Oncol Off J Am Soc Clin Oncol. (2023) 41:5163–73. doi: 10.1200/JCO.22.02630, PMID: 37647576 PMC10666986

[3] Conroy T Desseigne F Ychou M Bouché O Guimbaud R Bécouarn Y . FOLFIRINOX versus gemcitabine for metastatic pancreatic cancer. New Engl J Med. (2011) 364:1817–25. doi: 10.1056/NEJMoa1011923, PMID: 21561347

[4] Von Hoff DD Ervin T Arena FP Chiorean EG Infante J Moore M . Increased survival in pancreatic cancer with nab-paclitaxel plus gemcitabine. New Engl J Med. (2013) 369:1691–703. doi: 10.1056/NEJMoa1304369, PMID: 24131140 PMC4631139

[5] da Cunha Santos G Dhani N Tu D Chin K Ludkovski O Kamel-Reid S . Molecular predictors of outcome in a phase 3 study of gemcitabine and erlotinib therapy in patients with advanced pancreatic cancer: National Cancer Institute of Canada Clinical Trials Group Study PA. 3. Cancer. (2010) 116:5599–607. doi: 10.1002/cncr.25393, PMID: 20824720

[6] Philip PA Benedetti J Corless CL Wong R O’Reilly EM Flynn PJ . Phase III study comparing gemcitabine plus cetuximab versus gemcitabine in patients with advanced pancreatic adenocarcinoma: Southwest Oncology Group-directed intergroup trial S0205. J Clin Oncol Off J Am Soc Clin Oncol. (2010) 28:3605–10. doi: 10.1200/JCO.2009.25.7550, PMID: 20606093 PMC2917315

[7] André T Shiu KK Kim TW Jensen BV Jensen LH Punt C . Pembrolizumab in microsatellite-instability-high advanced colorectal cancer. New Engl J Med. (2020) 383:2207–18. doi: 10.1056/NEJMoa2017699, PMID: 33264544

[8] Marcus L Fashoyin-Aje LA Donoghue M Yuan M Rodriguez L Gallagher PS . FDA approval summary: pembrolizumab for the treatment of tumor mutational burden-high solid tumors. Clin Cancer research: an Off J Am Assoc Cancer Res. (2021) 27:4685–9. doi: 10.1158/1078-0432.CCR-21-0327, PMID: 34083238 PMC8416776

[9] Brahmer JR Tykodi SS Chow LQ Hwu WJ Topalian SL Hwu P . Safety and activity of anti-PD-L1 antibody in patients with advanced cancer. New Engl J Med. (2012) 366:2455–65. doi: 10.1056/NEJMoa1200694, PMID: 22658128 PMC3563263

[10] Weiss GJ Blaydorn L Beck J Bornemann-Kolatzki K Urnovitz H Schütz E . Phase Ib/II study of gemcitabine, nab-paclitaxel, and pembrolizumab in metastatic pancreatic adenocarcinoma. Investigational New Drugs. (2018) 36:96–102. doi: 10.1007/s10637-017-0525-1, PMID: 29119276

[11] Ullman NA Burchard PR Dunne RF Linehan DC . Immunologic strategies in pancreatic cancer: making cold tumors hot. J Clin Oncol Off J Am Soc Clin Oncol. (2022) 40:2789–805. doi: 10.1200/JCO.21.02616, PMID: 35839445 PMC9390820

[12] Nywening TM Wang-Gillam A Sanford DE Belt BA Panni RZ Cusworth BM . Targeting tumor-associated macrophages with CCR2 inhibition in combination with FOLFIRINOX in patients with borderline resectab le and locally advanced pancreatic cancer: a single-center, open-label, dose-finding, non-randomized, phase 1b trial. Lancet Oncol. (2016) 17:651–62. doi: 10.1016/S1470-2045(16)00078-4, PMID: 27055731 PMC5407285

[13] Cerniglia GJ Pore N Tsai JH Schultz S Mick R Choe R . Epidermal growth factor receptor inhibition modulates the microenvironment by vascular normalization to improve chemotherapy and radiotherapy efficacy. PloS One. (2009) 4:e6539. doi: 10.1371/journal.pone.0006539, PMID: 19657384 PMC2716529

[14] Garrido G Tikhomirov IA Rabasa A Yang E Gracia E Iznaga N . Bivalent binding by intermediate affinity of nimotuzumab: a contribution to explain antibody clinical profile. Cancer Biol Ther. (2011) 11:373–82. doi: 10.4161/cbt.11.4.14097, PMID: 21150278

[15] Schultheis B Reuter D Ebert MP Siveke J Kerkhoff A Berdel WE . Gemcitabine combined with the monoclonal antibody nimotuzumab is an active first-line regimen in KRAS wildtype patients with locally advanced or metastatic pancreatic cancer: a multicenter, randomized phase IIb study. Ann Oncol Off J Eur Soc Med Oncol. (2017) 28:2429–35. doi: 10.1093/annonc/mdx343, PMID: 28961832

[16] Pang X Huang Z Zhong T Zhang P Wang ZM Xia M . Cadonilimab, a tetravalent PD-1/CTLA-4 bispecific antibody with trans-binding and enhanced target binding avidity. mAbs. (2023) 15:2180794. doi: 10.1080/19420862.2023.2180794, PMID: 36872527 PMC10012886

[17] Gao X Xu N Li Z Shen L Ji K Zheng Z . Safety and antitumor activity of cadonilimab, an anti-PD-1/CTLA-4 bispecific antibody, for patients with advanced solid tumors (COMPASSION-03): a multicenter, open-label, phase 1b/2 trial. Lancet Oncol. (2023) 24:1134–46. doi: 10.1016/S1470-2045(23)00411-4, PMID: 37797632

[18] Wu X Sun Y Yang H Wang J Lou H Li D . Cadonilimab plus platinum-based chemotherapy with or without bevacizumab as first-line treatment for persistent, recurrent, or metastatic cervical cancer (COMPASSION-16): a randomized, double-blind, placebo-controlled phase 3 trial in China. Lancet (London England). (2024) 404:1668–76. doi: 10.1016/S0140-6736(24)02135-4, PMID: 39426385

[19] Wei SC Anang NAS Sharma R Andrews MC Reuben A Levine JH . Combination anti-CTLA-4 plus anti-PD-1 checkpoint blockade utilizes cellular mechanisms partially distinct from monotherapies. Proc Natl Acad Sci United States America. (2019) 116:22699–709. doi: 10.1073/pnas.1821218116, PMID: 31636208 PMC6842624

[20] Shapiro RM Sheffer M Booker MA Tolstorukov MY Ansuinelli M Sade-Feldman M . CTLA-4 blockade results in the enrichment of proliferative CD56 dimCD16 + NK cells following infusion of haploidentical donor memory-like natural killer cells plus IL-15 superagonist in a phase 1 trial. Blood. (2023) 142:6850. doi: 10.1182/blood-2023-178362

[21] Ho TTB Nasti A Seki A Komura T Inui H Kozaka T . Combination of gemcitabine and anti-PD-1 antibody enhances the anticancer effect of M1 macrophages and the Th1 response in a murine model of pancreatic cancer liver metastasis. J immunotherapy Cancer. (2020) 8. doi: 10.1136/jitc-2020-001367, PMID: 33188035 PMC7668383

[22] Reiss KA Mick R Teitelbaum U O’Hara M Schneider C Massa R . Niraparib plus nivolumab or niraparib plus ipilimumab in patients with platinum-sensitive advanced pancreatic cancer: a randomized, phase 1b/2 trial. Lancet Oncol. (2022) 23:1009–20. doi: 10.1016/S1470-2045(22)00369-2, PMID: 35810751 PMC9339497

